# A comparison between water uptake and root length density in winter wheat: effects of root density and rhizosphere properties

**DOI:** 10.1007/s11104-020-04530-3

**Published:** 2020-05-01

**Authors:** X. X. Zhang, P. A. Whalley, R. W. Ashton, J. Evans, M. J. Hawkesford, S. Griffiths, Z. D. Huang, H. Zhou, S. J. Mooney, W. R. Whalley

**Affiliations:** 1 Rothamsted Research, Harpenden, Hertfordshire AL5 2JQ UK; 2 University of Oxford, Radcliffe Observatory, Andrew Wiles Building, Woodstock Rd, Oxford, OX2 6GG UK; 3 John Innes Centre, Norwich Research Park, Norwich, NR4 7UH UK; 4 Farmland Irrigation Research Institute, Chinese Academy of Agricultural Sciences, Xinxiang, 453002 Henan China; 5 School of Biosciences, University of Nottingham, Sutton Bonington Campus, Loughborough, Leicestershire LE12 5RD UK; 6 State Key Laboratory of Soil and Sustainable Agriculture, Institute of Soil Sciences, Chinese Academy of Sciences, 71 East Beijing Road, Nanjing, 210008 People’s Republic of China

**Keywords:** Phenotyping, Soil water profile, Root length distribution, Simulation

## Abstract

**Background and aims:**

We aim to quantify the variation in root distribution in a set of 35 experimental wheat lines. We also compared the effect of variation in hydraulic properties of the rhizosphere on water uptake by roots.

**Methods:**

We measured the root length density and soil drying in 35 wheat lines in a field experiment. A 3D numerical model was used to predict soil drying profiles with the different root length distributions and compared with measured soil drying. The model was used to test different scenarios of the hydraulic properties of the rhizosphere.

**Results:**

We showed that wheat lines with no detectable differences in root length density can induce soil drying profiles with statistically significant differences. Our data confirmed that a root length density of at least 1 cm/cm^3^ is needed to drain all the available water in soil. In surface layers where the root length density was far greater than 1 cm/cm^3^ water uptake was independent of rooting density due to competition for water. However, in deeper layers where root length density was less than 1 cm/cm^3^, water uptake by roots was proportional to root density.

**Conclusion:**

In a set of wheat lines with no detectable differences in the root length density we found significant differences in water uptake. This may be because small differences in root density at depth can result in larger differences in water uptake or that the hydraulic properties of the rhizosphere can greatly affect water uptake.

**Electronic supplementary material:**

The online version of this article (10.1007/s11104-020-04530-3) contains supplementary material, which is available to authorized users.

## Introduction

The yield of wheat is closely related to the ability of its roots to access water (Passioura 1983); however, wheat roots are not effective at capturing the available water, even at relatively shallow depths (Gregory et al. 1978a, b; Hodgkinson et al. 2017; Ober et al. 2014). Wheat lines with an improved ability to capture water in deeper layers have been linked to higher yields (Ober et al. 2014; Lilley and Kirkegaard 2011). The inability of roots to access water is commonly attributed to a low root length density at depth (Gregory et al. 1978a, b). For this reason, rooting depth of wheat in the UK, and elsewhere, has been of considerable interest (e.g. Lupton et al. 1974; Gregory et al. 1978a; Barraclough and Leigh 1984; White et al. 2015; Ober et al. 2014; Wasson et al. 2014; Hodgkinson et al. 2017). However, within the wheat population that is currently grown commercially in the UK, there does not appear to be strong evidence for large differences in deep rooting (Bai et al. 2019; Hodgkinson et al. 2017; White et al. 2015). Despite this there is recent evidence that some lines are more effective at accessing deep water than others (Ober et al. 2014; Whalley et al. 2017). A possible explanation for differences in water uptake despite similar distributions in rooting depth is differences in soil hydraulic properties due to root exudation. One effect of microbial stimulation by root exudates is to greatly reduce water uptake by roots and water flow in soil (Choudhury et al. 2018; Zarebanadkouki et al. 2018). Kroener et al. (2016) have shown that the hydraulic properties of the rhizosphere can greatly affect water uptake by roots and the reduced conductivity of the rhizosphere soil to water can reduce the effects of drought stress. It is also possible that the differences in root hair number and length might explain the differences in water uptake by roots (e.g. Carminati et al. 2017) between wheat lines with similar root length distributions. To explore these effects, we used a numerical model to elucidate how root length distribution of 35 lines modulate water uptake from the soil profile. Unlike the one-dimensional macroscopic numerical models that treat the root water uptake as a sink by averaging the delicate water pressure and root architecture in the horizonal direction (e.g., Cai et al. 2017, 2018), our model is three dimensional with all root segments explicitly represented in attempts to capture their competition for water.

The potential to use spatial patterns in soil water to infer root activity has been recognised for sometime. For example, Nelson et al. (2006) found that soil water content measurements had the potential to be used to map the vertical and horizontal root spatial activity in oil palm trees. Moroke et al. (2005) showed that soil water content profiles were related to root length density in cowpea, sorghum and sunflower. They used soil water content measurements to indicate rooting depth. In a recent comparison of five wheat lines, Hodgkinson et al. (2017) showed that when significant differences in root length distributions were recorded, there were also significant differences in soil drying, determined with a neutron probe. However, Wasson et al. (2012) suggest that the indirect root phenotyping should be used with some caution, arguing that comparisons of soil drying profiles can be confounded by the field site and the weather in the year of testing. An extreme example is that of deep rooted plants which have roots below the groundwater table due to heavy rain, where the roots have been rendered inactive, compared to a shallow rooted plant. Nevertheless, indirect phenotyping with soil moisture measurement has important advantages, not least that it has the potential to be rapid (Whalley et al. 2017).

This paper has two objectives. Firstly, we report on a comparison of root length distributions with depth and soil water uptake in 35 near isogenic wheat lines. To help interpret these data we used a model of root water uptake to predict water uptake as a function of depth using measured root length data. Once validated against field data, the model was used for our second objective, to investigate the effects of the changes in hydraulic properties of soil adjacent to the root, such as those caused by root exudation, on root water uptake. This is important because differences in the hydraulic properties of the rhizosphere are a possible explanation for difference in water uptake between wheats with similar root length distributions. The model also enables us to explore the effect of seasonal differences on soil drying, by using different initial soil water profiles. Experimental studies (Whalley et al. 2017) suggest that whether or not soil drying patterns reveal significant differences between wheat lines may in part depend on seasonal differences in rainfall. Here we will test the impact of different rainfall patterns on the same root length distribution with depth.

## Materials and methods

### Field experiment

The experiments were conducted on Broadmead Field at Woburn Experimental Farm, Bedfordshire, UK (52°01′11.2”N 0°35′30.4”W). In this field, soil in the 0 to 40 cm layer was a silt-clay loam. There is a vertical gradient in texture in the depth of 1 m. Deeper layer has a greater sand content (Hodgkinson et al. 2017). The surface layer (approximately 30 cm) has more organic matter content. To the depth of 60 cm the bulk density of the soil does not change greatly, and it is approximately 1.2 g/cm^3^. Soil properties are summarized in Table 1. The soil profile on Broadmead is consistent with the description of a soil profile by Weir et al. (1984) that should be expected to produce high yields of winter wheat.

**Table 1:** Description of the topsoil (0 to 40 cm below the surface) properties of Woburn Experimental Field Station, Bedfordshire, UK

Property	Units	
Location	Latitude	52°01′06”N
	Longitude	00°35′30”W
Soil type	SSEW group	Typical alluvial Gley soil
	SSEW series	Eversley
	FAO	Fluvisol
Sand (2000–65 μm) Surface soil	g g^−1^ dry soil	0.538
Silt (63–2 μm)	g g^−1^ dry soil	0.203
Clay (< 2 μm)	g g^−1^ dry soil	0.260
Texture	SSEW class	Sandy clay loam
Particle density	g cm^−3^	2.587
Organic matter	g g^−1^ dry soil	0.038

The field experiment had 504 separate 9 m × 1.8 m plots, divided into three fully randomised blocks, with each block containing 168 plots of different wheat lines and one fallow plot.

Thirty-five near isogenic wheat lines (see supplemental data) were randomly arranged within each block. The plots were sown on 10/10/2017. The field site was rain-fed with no additional irrigation. Husbandry of the crops followed standard agronomic protocols for the UK, with inputs to ensure adequate nutrition, weed, and pest and disease control.

Water content profiles were measured in the growth season at four-time points starting on 05/03/2018, 18/04/2018, 15/05/2018 and 11/06/2018. To measure the soil water content profiles on all 246 plots, it took 4 days with a Neutron probe (CPN HydroProbe model 503TDR). Aluminium access tubes were installed approximately 1 m from the end of the selected plots and measurements were made at depths of 0.15, 0.30, 0.50, 0.70, 1.00, 1.25 and 1.45 m.

Leaf area index was measured on 17/04/18, 18/05/18 and 13/06/18 with a Delta-T Sun-scan ® (Delta-T Devices, Burwell, Cambridge, UK).

### Soil cores to estimate root distribution

Cylindrical soil cores were taken from the Broadmead plots between 4/7/18 and 2/8/18 using a soil column cylinder auger (Van Walt Ltd., Surrey, UK). The cores were 100 cm long and 5 cm in diameter. They were extracted approximately 1 m from the end of the plots and at the end opposite to the neutron probe access tube. Once extracted, the soil cores were stored inside a black plastic bag. We took one core from each plot for the 35 lines (see supplemental information). All three blocks were sampled to give a total of 105 soil cores. All these plots were also monitored with a neutron probe, as outlined above, to give data on both root length distribution and soil drying.

We used the core break method to estimate root length distributions with depth (Hodgkinson et al. 2017). These cores were then broken approximately 5 cm from the top of the core to reveal fresh faces, exactly as described by White and Kirkegaard (2010), and then every 10 cm. On each face the number of roots was counted three times, but the core was rotated 120° between successive counts. The number of roots at each break point is the summation of roots on both sides of the break point. Root count data were converted into root length density data assuming the roots were parallel along the axis of the core. The core break method correlates well with the root washing approach (Wasson et al. 2014) and is more convenient when a large number of cores are involved, as in this study.

### A model for root water uptake

We simulated water flow and the associated root uptake by explicitly representing the extracted root segments using the following 3D Richards’ equation:

1 $$ \frac{\mathrm{\partial \uptheta }}{\partial t}=\nabla \boldsymbol{K}\nabla \left(h-z\right), $$

where *θ* is volumetric water content, *K* is hydraulic conductivity, *h* is matric potential and z is soil depth.

**Fig. 1:**
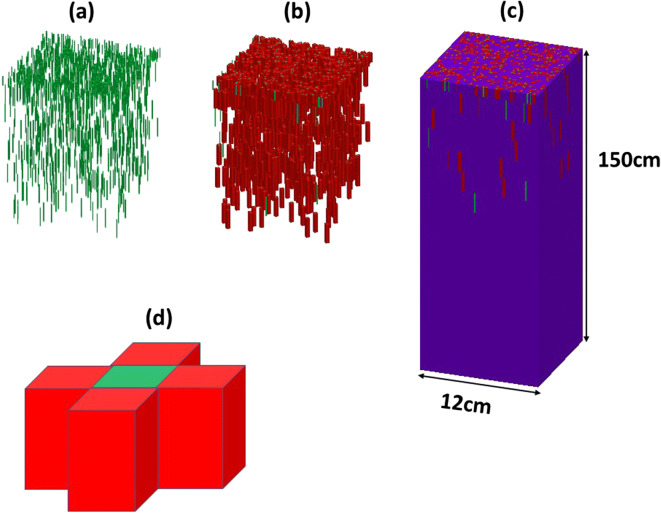
Distribution of the measured root segments in soil was stochastically generated (**a**). Each root segment was associated with a rhizosphere 2.5 mm thick (**b**). Soil in other area was treated as bulk soil, and the soil column was then divided into 120 × 120 × 300 cuboids for numerical simulation, each being 1 mm × 1 mm × 5 mm (**c**). An illustrative example showing one root cuboid with its four bordered rhizosphere cuboids through their interfaces water flows from soil into the root (**d**)

The soil column in numerical simulation for all treatments was 12 cm ×12 cm in horizontal cross section and 150 cm in depth, and it was discretized into 120 × 120 × 300 cuboid elements, each being 0.1 cm × 0.1 cm × 0.5 cm. To be consistent with the measured root density, root distribution in the soil column was stochastically generated under the constraint that the root-length density at different depth generated stochastically was the same as the measurement. In the stochastic generation, the location of each segment was assumed to be independent of each other. Figure 1 shows an exemplary root segment distribution constructed by the stochastic model.

The above equation was solved using a 3D volume element method based on the scheme of Celia et al. (1990), and the distribution of the root segments was explicitly resolved by treating the water-root interface as a boundary. At the interface between a root element (cuboid i) and a soil element (cuboid j) in the volume element method, water flow rate (i.e., root uptake rate) from the soil element to its adjacent root element was calculated as follows:

2 $$ {q}_{i,j}=\frac{1}{\delta x}\frac{2{K}_{soil}{K}_{root}}{K_{soil}+{K}_{root}}\left[\left({h}_i+{z}_i\right)-\left({h}_j+{z}_j\right)\right],\kern1em , $$

where *K*_*soil*_ and *K*_*root*_ are hydraulic conductivity of the soil element and the radial hydraulic conductivity of the root element respectively, *δ*_*x*_ is the distance between the centres of the soil and the root elements, *h*_*i*_ and *h*_*j*_ are water pressure head in the soil element and the root element respectively, and *z*_*i*_ and *z*_*j*_ are the associated depth of the centre of the two elements. Since the resistance to water flow in the root is dominated by radial hydraulic resistance, we assumed that the axial resistance was comparably negligible and water pressure inside all root segments was thus the same and remained unchanged during the simulation. Mathematically, this was equivalent to treating the water-root interface as a first-type boundary where the matric potential was the water pressure head inside the xylem and was specified. Spatially discretizing the Richards’ equation in the numerical model resulted in a non-linear system with 4.32 million cuboid elements with each cuboid element having a matric potential defined in it. The non-linearity was solved using the iterative method we proposed previously (Zhang et al. 2002) and the linearized system during the iteration was solved using the preconditioned conjugate gradient method (Van der Vorst 1992). The simulation time for all treatments was 30 days and during the simulation, we calculated the water flow rate from soil to root at each soil-root element interface. The overall root water uptake from a soil layer is the summation of the water flow rates through all soil-root element interfaces in this layer. To be consistent with the experiment, we calculated water uptake for each of the following layers: 0–5, 5–15, 15–25, 25–35, 35–45, 45–55, 55–65, 65–75, 75–85, 85–95 cm. Further, we estimated changes in water content at 35, 55, 75, and 95 cm by interpolation, to provide comparisons between measured root length density, changes in water content and simulated root water uptake. Observed data revealed that soil moisture at the depth of 100 cm remained almost unchanged and the bottom of the column  was thus treated as a free-drainage boundary where the gradient of matric potential was zero; the soil surface was treated as no-flow boundary.

### Impact of the rhizosphere

The effect of the rhizosphere on root water uptake was simulated by differentiating the hydraulic properties of the rhizosphere from the bulk soil. There is a consensus in the literature that the rhizosphere of each root is the 1–3 mm of soil surrounding it. In all simulations, we assumed that the thickness of the rhizospheres was 2.5 mm and numerically constructed the model based on the stochastically constructed root distribution by coating a rhizosphere over each root segment as shown in Fig. 1. With the rhizosphere explicitly represented, the root water uptake occurring at the interface of root and the rhizosphere was calculated in the numerical model using Eq. (2) with the soil hydraulic properties replaced by the rhizosphere hydraulic properties. In all rhizosphere scenarios we considered, the bulk soil was assumed to be hydraulically identical and only the rhizosphere properties changed as shown in Table 2.

**Table 2:** Hydraulic parameters of the rhizosphere at the three scenarios. We give the parameters of the van Genuchten equation (θ_s_, θ_r_ α and n) and the hydraulic conductivity (K_s_) of the saturated soil

	θ_s_	θ_r_	α (cm^−1^)	n	K_s_(cm day^−1^)
Case i (hydrophobic soil)	0.55	0.01	2.4	1.0775	400
Case ii (compacted rhizosphere)	0.50	0.012	0.98	1.07	350
Case iii (loose rhizosphere)	0.59	0.008	1.31	1.098	450
Control (no rhizosphere)	0.55	0.01	1.5	1.08	400

**Fig. 2:**
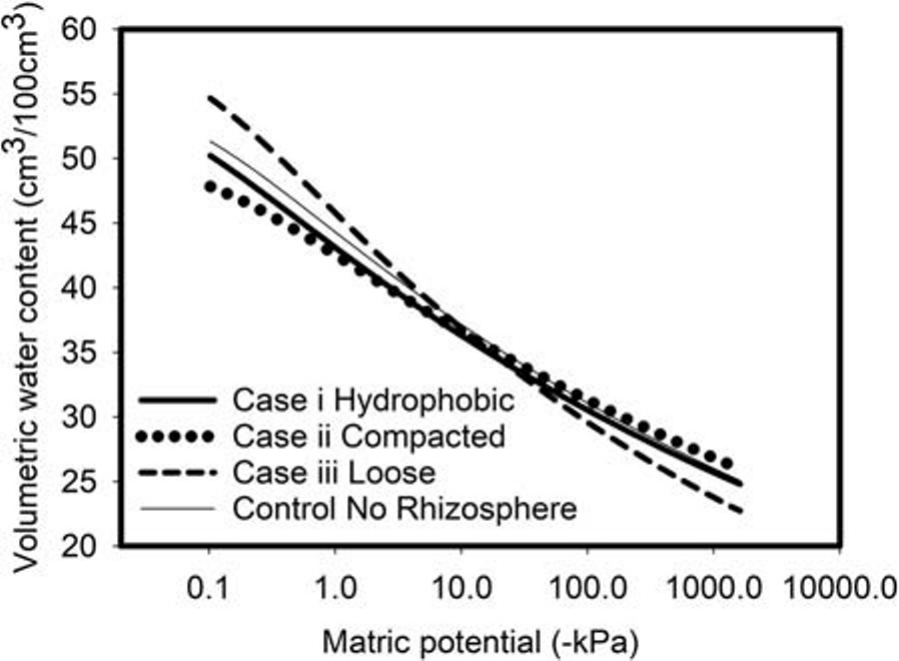
Three different water release scenarios used to explore the effects of rhizosphere soil on root water uptake. The van Genuchten parameter values are given in Table 2 along with the saturated hydraulic conductivities. These water releases represent the changes due to root activity relative to the bulk soil (also shown). Case i represents a hydrophobic rhizosphere, case ii represents a compacted rhizosphere while case iii represents a loose rhizosphere. The water release curve in the absence of a modified rhizosphere is also shown, called “no rhizosphere” where the bulk soil has the same hydraulic properties as the soil adjacent to the root

We considered three mechanisms that could cause physical change in the rhizosphere and compared them, even though in the field they are likely to work interactively to reshape the rhizosphere. The first one was based on the work of Read et al. (2003) that the root exudates rendered the rhizosphere hydrophobic while the soil structure and saturated hydraulic conductivity remained unchanged. The second one was based on the study of Aravena et al. (2011) that root growth compacted the rhizosphere, for which we assumed that the compaction was due to the compression of large pores leading to an increase in volume of small pores and reduction in saturated hydraulic conductivity. The third one was based on the recent work of Helliwell et al. (2017) and Rabbi et al. (2018) that root growth and its associated activities increased the porosity of the rhizosphere due to root thigmotropism and the increased aggregation, which led to a reduction in volume of small pores and an increase in saturated hydraulic conductivity as shown in Rabbi et al. (2018). The control was “no rhizosphere” in that the hydraulic properties of the rhizosphere and the bulk soil were the same. Along with hydraulic properties of the bulk soil, the hydraulic parameters of the rhizosphere associated with each scenario are given in Table 2 and Fig. 2.

There were several rainfall events in the early growth stage. As there were no measurements of surface runoff associated with each rainfall, we only simulated the growth stage without rainfall from May to June to avoid uncertainty induced by rainfalls and to be consistent with the time that the root density measurements were taken. The simulation was to reproduce the measurement starting from 13/05/2018 to 17/05/2018 for different blocks depending on when the soil moisture measurement was taken from it and running for approximately 30 days when the second measurement was taken from each block. During the simulation, we sampled the root uptake rates at all root-soil element interfaces as shown in Fig. 1. They were then averaged across each horizontal section to calculate the average root water uptake along the profile and summed up to calculate the accumulative change in the transpiration. For comparison with the measurement, we averaged the simulated root uptake rate for each of the ten layers over the simulation period.

### Statistical analysis

Root counts were analysed with a linear mixed model and were fitted using REML (residual, maximum likelihood) with the statistics package Genstat 19 (VSNI, 2 Amberside House, Wood Lane, Hemel Hempstead, HP2 4TP, England UK). The fixed structure was wheat line* depth, i.e., the line fitted over depth for each variety had a different slope and intercept. The random structure was Block/Plot/Depth/face/rotation where the face and rotation represent pseudo replicates of the samples at each depth. The covariance structure included for depth was an auto-regressive process of order 1. This accounted for the possibility that samples at adjacent depths were likely to be correlated. A cubic spline term was included over depth to capture non-linear departures from the model. This spline term was common to all varieties. A square root transformation was required to satisfy the assumption that variance is independent of the mean.

Soil drying was analysed with a linear mixed model and was fitted using REML in Genstat 19. The fixed structure included main effects of variety, measurement time and depth. Two-way interactions wheat line*depth and time*depth were also included i.e. the line fitted over depth for each variety and for each time had a different slope and intercept. This fixed model was selected using a backward selection process. The random structure was Block/Plot/sample/subsample where each sample corresponds to a measurement time and each subsample corresponds to a depth. A cubic spline term was included over depth to capture non-linear departures from the model. A separate spline term was included for each variety and time i.e. the departures from the model were different for each variety and time combination.

The simulated root water uptake data estimated from the mean of the measured root length density data at each depth for each plot was assessed with analysis of variance (ANOVA). The treatment structure was wheat line*depth with a block structure of block/plot/depth.

## Results

**Fig. 3:**
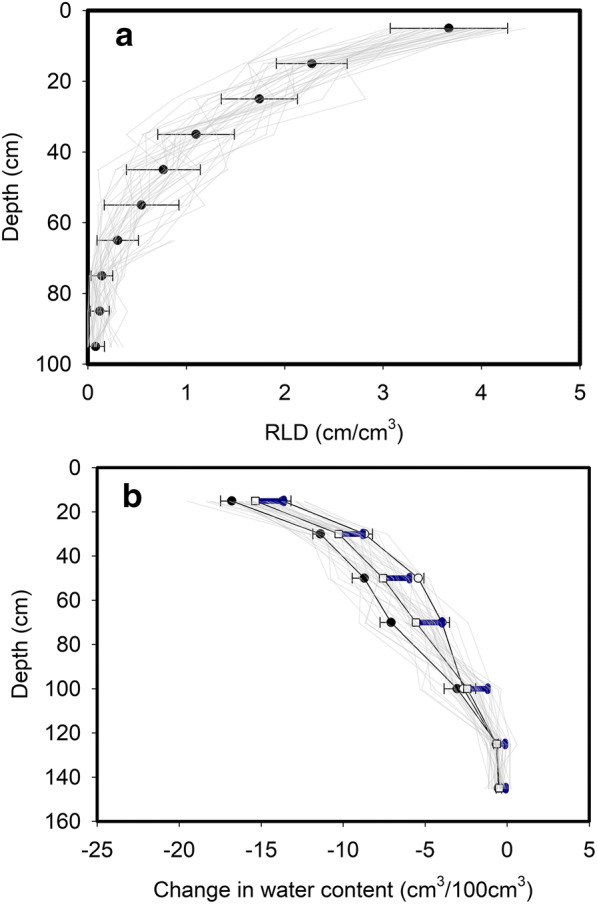
Panel A shows the mean root length density (RLD) estimated with the core break method plotted against depth. The standard deviations are indicated. The main effects of wheat line and depth were significant at *P* = 0.249 and *P* < 0.001 respectively. The effect of the interaction between wheat line and depth was not significant (*P* = 0.972). Panel B shows the change in volumetric water content plotted against depth. The mean values are plotted, and the standard deviations are indicated (open squares and blue error bar). The main effects of wheat line, time and depth were significant at *P* = 0.592, *P* = <0.001 and *P* < 0.001 respectively. The effect of the interaction between wheat line and depth was also significant (*P* < 0.001) and the interaction between wheat line, depth and time was not significant (*P* = 0.346). Also plotted in panel B are the mean changes in water content as a function of depth for the five lines with the greatest amount of soil drying, based on the total soil drying (solid circles) and the five lines with the least amount of soil drying (open circles).The error bars indicate the standard error

We did not find any significant interactions between the wheat line and depth (Fig. 3a; *P* = 0.972) with respect to root length density, nor was the main effect of the wheat line on root count significant (*P* = 0.249). These data do not support any genotypic differences in root depth between these wheat lines. Figure S1 shows the rainfall pattern during 2018 and the potential soil moisture deficit. The first and final time points that soil water contents were measured are marked on Fig. S1 and the change in soil water content between these time points is shown in Fig. 3b. Analysis of the soil moisture data with REML showed that these wheat lines had significantly different water uptake patterns both in time and with depth. In Fig. 3b we have plotted the mean change in the water content as a function of depth for 5 NILs with the greatest and the least amount of soil drying. At depths of 55 and 75 cm root length density and soil drying have the same rank order (*P* = 0.042 and *P* = 0.009, respectively; Table 3).

**Table 3:** Comparison of the rank order of 35 wheat lines root length density and modelled soil drying (change in soil water content) at five depths using the Spearman’s rank correlation coefficient

Depth (cm)	Correlation coefficient	*P*
15	−0.185	0.072
35	0.135	0.111
55	0.238	0.042
75	0.353	0.009
95	−0.118	0.110

For all the 35 lines, we analysed the relationship between root-length density and change in water content, which was simulated over the 30 days. Since the length of the cuboid in numerical simulation was 0.5 cm, to be consistent with the dimensions of the auger (5 cm in diameter and 100 cm long) used to measure the root-length density, the water content change was the average across the lateral cross section and over depth which was consistent with the measured data from the neutron probe. In some regions (mainly subsoil) without root there is a slight increase in water content due to vertical water flow, while for areas with higher root density, the soil water content had decreased. The change in water content varied for the same root-length density, and such variations are amplified as root density increased because of increase in root competition for water. How much water the roots could take up under such conditions depend on lateral distribution of the roots as well as the availability of water in adjacent areas, which varies from line to line and plot to plot. Overall, the “grouped” water content changes increased linearly with “grouped” root density although their relationship for individual line in individual plot is non-linear. We simulated the impact of three different initial water contents on root water uptake of all lines: the measured soil water profile on 05/03/2018, a wet soil profile and a dry soil profile. Figure S2 shows the non-linear relationship between the simulated root water uptake rate and the root length density for the three scenarios. The simulated data from the initially wet soil profiles form an upper boundary for the relationships in Fig. S2C. For the data set, the water uptake rate (q) can be related to root density (S) by

3 $$ q=a\left(1-{e}^{- bS}\right) $$

As S is large in the surface layers the rate of root water uptake is constant and does not vary greatly with S and *q* ≈ *a* which is the maximum rate of water uptake by roots. For a given value *a*, the *b* parameter determines how quickly *q* increases with S. Analysis of variance on the parameters fitted to Eq. 3 showed that *a* was not significantly affected by line while *b* was significantly affected by the wheat line at *P* = 0.061. However, grouped non-linear regression showed that each line was defined by a unique relationship between *q* and *S* (*P* < 0.001). Figure S2A are data for the measured change in water content and also plotted against root length density. These data are best described by linear regression and grouped regression showed that these data were best described with a common intercept and a slope that depended on wheat line (*P* = 0.033).

To assess the reliability of the model at describing the field data, we compared the change in soil water content measured in the field with the root water uptake predicted by the model (Fig. S3) and found good agreement. Rank correlations between modelled root water uptake and the measured change in soil water content, at different depths, provided further confirmation that the modelled root water uptake reflected the field data (Table 4) at depths between 35 and 75 cm. In Fig. S3 we plotted data for the different depths averaged over the different lines. We assumed that there was no difference in the water demanded by the shoots when running the model because REML analysis showed that there was no significant effect of wheat line on leaf area index (*P* = 0.209) nor was there any significant interaction between measurement date and leaf area index (*P* = 0.976). Only measurement date had a significant effect on leaf area index (*P* < 0.001).

**Table 4:** Comparison of the rank order of 35 wheat lines according modelled root water uptake and measured soil drying (change in soil water content) at five depths using the Spearman’s rank correlation coefficient

Depth (cm)	Correlation coefficient	*P*
15.	0.183	0.073
35	0.397	0.005
55	0.513	<0.001
75	0.439	0.002
95	−0.108	0.123

**Fig. 4:**
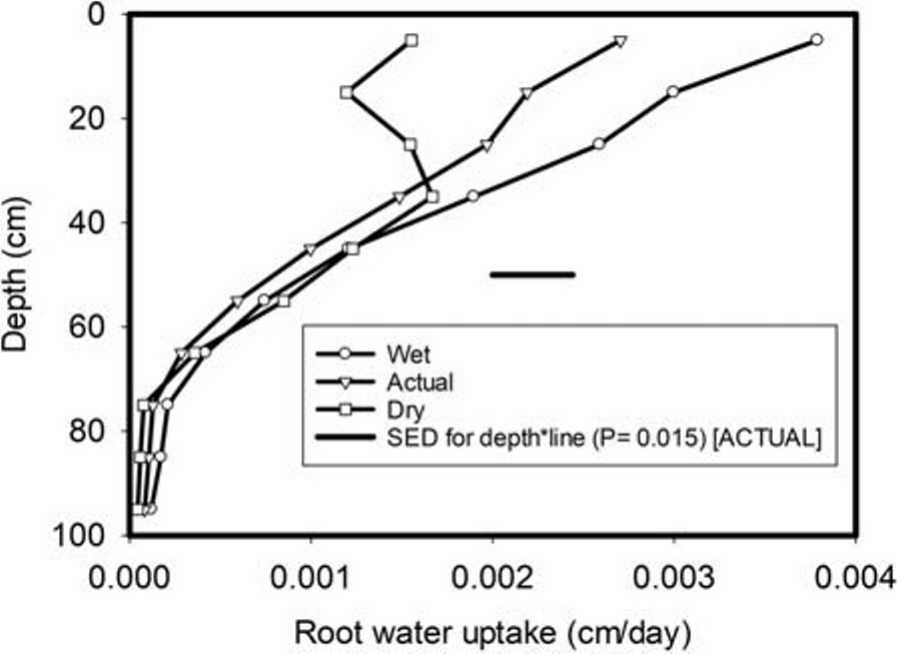
Predicted root water uptake as function of depth using the model illustrated in Fig. 1 and the root length distribution data in Fig. 3a. Three different initial soil water profiles were used: the measured soil water profile, a wet soil water profile and a dry soil water profile. The main effect of depth was significant in all three cases (*P* < 0.001). The main effect of wheat line was not significant at *P* = 0.548, *P* = 0.304 and *P* = 0.504 for the initially wet, the actual and the initial dry profiles respectively. The effect of the interaction between wheat line and depth was significant at *P* = 0.504, *P* = 0.015 and *P* = 0.332 for the initially wet, the actual and the initial dry profiles respectively

When the simulations were made using the measured soil water content for each line as a starting point, ANOVA analysis of the predicted water uptake rate showed a significant effect of depth (*P* < 0.001) as well as a significant interaction between root water uptake rate and depth (*P* = 0.015), but the effect of wheat line (*P* = 0.304) was not significant (Fig. 4). In the simulation made with an initially wet and dry soil profile, only depth had a significant effect (*P* < 0.001) on the simulated root water uptake data and there were no statistically significant interactions between wheat line and depth.

**Fig. 5:**
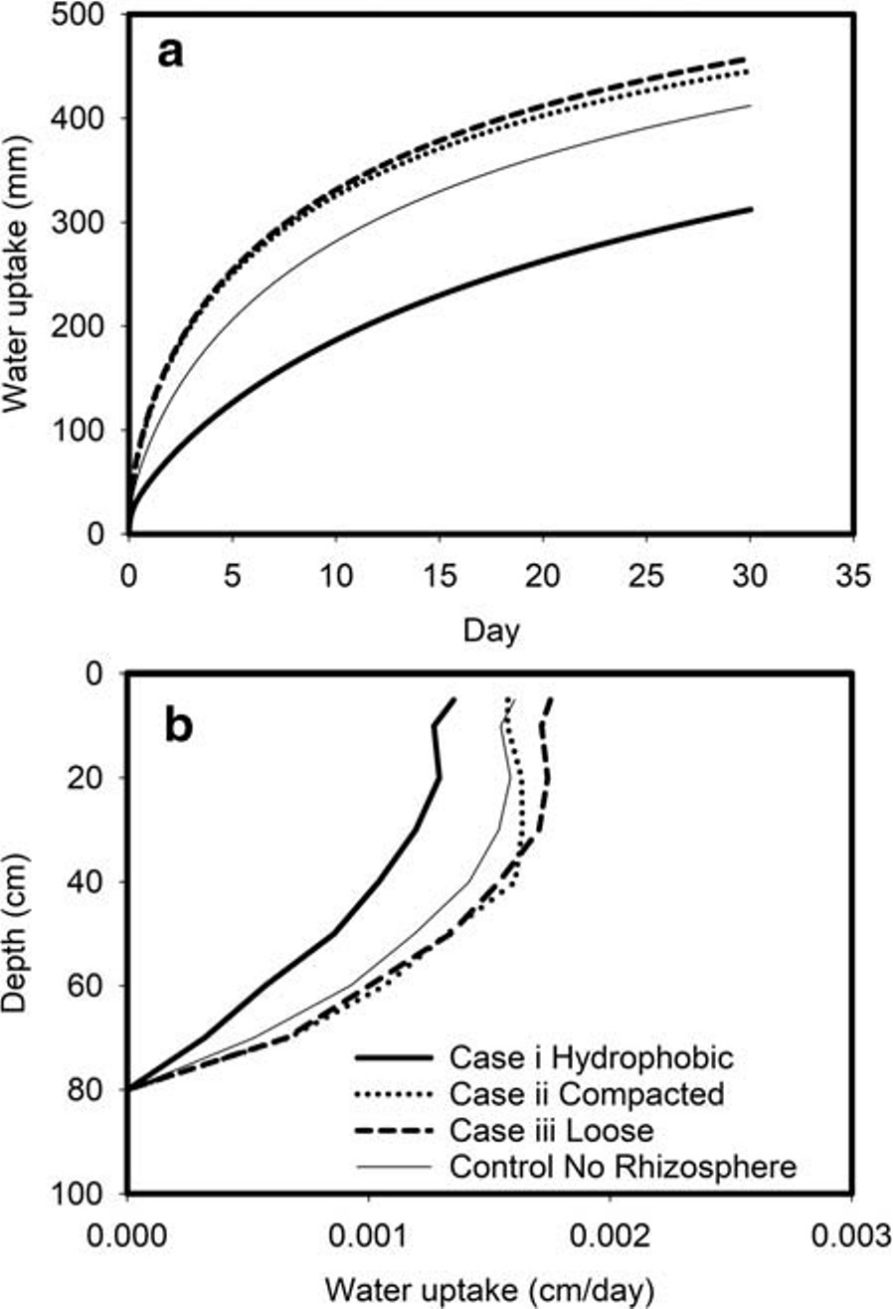
Panel A shows predicted root water uptake as a function of time for roots with three different rhizospheres. Case i represents a hydrophobic rhizosphere, case ii represents a compacted rhizosphere while case iii represents a loose rhizosphere. The water uptake profile in the absence of a modified rhizosphere is also shown, called “no rhizosphere” where the bulk soil has the same hydraulic properties as the soil adjacent to the root. Panel B shows predicted root water uptake as a function of depth for roots with three different rhizospheres. The water uptake profile in the absence of a modified rhizosphere is also shown. Case i represents a hydrophobic rhizosphere, case ii represents a compacted rhizosphere while case iii represents a loose rhizosphere

Simulations of the effect of a potential modification to the hydraulic properties of the rhizosphere soil (Table 2; Fig. 2) are shown in Fig. 5. A change in the porosity of the rhizosphere increased water uptake (case ii and iii) while a hydrophobic rhizosphere (case i) decreased water uptake.

## Discussion

Analysis of the data for root length density implied that significant differences in the root length distributions with depth or total quantity of roots could not really be detected (Fig. 3). However, the soil drying data showed the effects of wheat line are significantly different from the same set of wheats (*P* < 0.001, Fig. 3). Since leaf area index, in this collection of 35 lines did not differ significantly between these wheats (*P* = 0.209) nor was there interaction between leaf area index and time (*P* = 0.976), we assume that the differences in soil drying with depth were primarily related either to differences in root length distributions or their effectiveness at water uptake. When measured root length data was used to predict root water uptake, we found significant effects of depth (*P* < 0.001) as well as a significant interaction (*P* = 0.015) in the simulations. The consistency in the rank correlation between modelled root water uptake and the measured changes in water content provides some assurance that the model accurately simulates soil drying by the different wheat lines. While the different relationships between root water uptake and root length density for the wheat lines could not be simply related to the two parameters in Eq. 3, grouped regression for each line did show that these relationships were significantly different (*P* < 0.001). These differences could be either due to root permeability or differences in how roots modify rhizosphere soil (Helliwell et al. 2019). It should be noted that the linear relationship between measured soil drying (change in water content) and root length density shown in Fig. S2A did not account for water flow because the matric potential gradient generated by root water uptake could move water from root-poor areas into root-rich areas. As such, significant amounts of soil drying could occur at very low root length densities due to the redistribution of water in the soil profile which was taken into account in the model but not in the measured soil water content data. In Fig. S2B which shows the modelled change in water content that is only due to root water uptake, the change in water content at low root length densities is small. We used root length density data (Fig. 3a) to estimate root water uptake plotted in Fig. 4 for all 10 layers and 35 wheat lines, using the simulation model. Distribution of root segments is spatially heterogeneous and water flow induced by root uptake makes water content vary both vertically and laterally in the representative column in Fig. 1. If in a lateral section where the roots are clustered, continued water uptake dried the soil thereby leading to root competition for water. Therefore, even when root length density was the same at a depth, its water uptake might vary widely depending on how these roots distribute in the soil.

In surface (15 cm) and very deep layers (95 cm) there was no significant rank correlation (Table 3) between the measured root length density and soil drying. In the surface layer, the root density was so high that all wheats could dry the soil (Gregory et al. 1978a, b), while in deep layers there are too few roots to enable soil drying. This is similar to the finding of Zhang et al. (2004) that wheat took most water from the shallow soil although its roots can grow down to 200 cm deep. Drying at intermediate depths (70 to 110 cm) has been found to be positively correlated with water uptake and yield (Ober et al. 2014). In previous work, we found that the number of roots deeper than 45 cm was positively correlated with grain yield and explained 13% of yield (*P* < 0.001) in a dry season, but only 4% (*P* = 0.045) in a wet season (Bai et al. 2019). At depths deeper than approximately 55 cm, where root length density is much smaller than 1 cm/cm^3^, water uptake is a function of root length density, and increased root length density provides increased water uptake. In contrast, the correlation between water uptake and root length density in surface layers is much weaker (Fig. S4), due to the increased competition for water between roots. In our simulations, we found that the effect of our different root distributions on root water uptake was significant when the measured soil water profile was used in the simulation but not when the dry or wet soil profiles were used (i.e. depth*wheat line interaction of *P* = 0.015 using the measured but *P* = 0.504 in a wet profile and *P* = 0.332 on a dry profile). The implication of this is that significant differences in root water uptake, for a given set of root profiles, are unlikely to be found in the extremes of wet years or dry years. Although, it is likely that the season will greatly affect the root length distribution (e.g. Hodgkinson et al. 2017).

Root growth alters the hydraulic properties of the rhizosphere soil (Whalley et al. 2004, 2005), but there is no consensus on how it physically reshapes soil structure in the rhizosphere. Increase in the porosity of rhizosphere soil has been reported (Rabbi et al. 2018; Helliwell et al. 2017, 2019) while others showed a decrease (Braunack and Freebairn 1988). It has also been shown that root exudations could make the rhizosphere hydrophobic (Read et al. 2003; Cooper et al. 2018; Whalley et al. 2004). While it is likely these processes may occur concurrently in soil, to differentiate how each change affects root uptake from different locations in soil, we simulated three scenarios. Simulated water flow and root uptake processes for 30 days (Fig. 5a, b) show that physical modification to the rhizosphere, due to an increase or decrease in porosity, increased water uptake while the effect of a hydrophobic rhizosphere was to reduce water uptake. It appears that the effect of modification to the rhizosphere by roots, either by a change in porosity or a change to its hydrophilic status, might be as great as or greater than a change in root length distribution. At present it is unknown if different wheat lines have different effects on soil structure in the rhizosphere, although there is evidence that barley and wheat can have different effects on the water release characteristic of the rhizosphere (Whalley et al. 2005).

To summarise, on a set of 35 wheat lines we found little evidence of any differences in root length distribution with depth while significant difference in water uptake by roots were observed. There are three possible explanations:

1. Differences in root density needed to provide significant differences in soil drying are too small to be detected with the root count method.
2. Differences in the spatial distribution of roots account for differences in water uptake
3. Different wheat lines are associated with rhizospheres with different hydraulic properties, due to the effects of root exudates or differences in soil structure.

## Conclusions

Statistical analysis of root length distribution data indicates limited genotypic differences between the wheat lines we studied. Numerical analysis of water uptake showed that in the surface densely rooted layer (root length density > 1 cm/cm^3^) there was only a weak relationship between water uptake and root length density while at depth (when root length density < 1 cm/cm^3^) water uptake is proportional to root length density. Prediction of root water uptake from a set of root length data with statistically similar depth profiles (i.e. *P* > 0.05) showed statistically significant interactions between wheat line and depth. The effect of genotype on soil water uptake is almost certainly due to the difference in root length density in deeper layers where water uptake is proportional to root length density. Numerical analysis showed that differences in rhizosphere soil structure increased water uptake irrespective of the assumptions made about rhizosphere soil (i.e. whether it was more or less dense). The assumption of a hydrophobic rhizosphere greatly reduced water uptake.

## Electronic supplementary material

ESM 1 (DOCX 961 kb)

## References

[CR1] Aravena JE, Berli M, Ghezzehei TA, Tyler SW (2011). Effects of root-induced compaction on rhizosphere hydraulic properties - X-ray microtomography imaging and numerical simulations. Environ Sci Technol.

[CR2] Bai C, Ge Y, Ashton RW, Evans J, Milne A, Hawkesford MJ, Whalley WR, Parry MAJ, Melichar J, Feuerhelm D, Bansept Basler P, Bartsch M (2019). The relationships between seedling root screens, root growth in the field and grain yield for wheat. Plant Soil.

[CR3] Barraclough PB, Leigh R (1984). The growth and activity of winter wheat roots in the field: the effect of sowing date and soil type on root growth of high yielding crops. J Agric Sci.

[CR4] Braunack MVA, Freebairn DM (1988). The effect of bulk density on root growth. Proceedings of the 11th International Conference of the International Soil Tillage Research Organisation, Volume I.

[CR5] Cai GC, Vanderborght J, Couvreur V, Mboh CM, Vereecken C (2017). Parameterization of root water uptake models considering dynamic root distributions and water uptake compensation. Vadose Zone J.

[CR6] Cai GC, Vanderborght J, Langensiepen M, Schnepf A, Hüging H, Vereecken H (2018). Root growth, water uptake, and sap flow of winter wheat in response to different soil water conditions. Hydrol Earth Syst Sci.

[CR7] Carminati A, Passioura JB, Zarebanadkouki M, Ahmed MA, Ryan PR, Watt M, Delhaize E (2017). Root hairs enable high transpiration rates in drying soils. New Phytol.

[CR8] Celia MA, Bouloutas ET, Zarba RL (1990). A general mass-conservative numerical-solution for the unsaturated flow equation. Water Resour Res.

[CR9] Choudhury BU, Ferraris S, Ashton RW, Powlson DS, Whalley WR (2018). The effect of microbial activity on soil water diffusivity, implications for water uptake by roots. Eur J Soil Sci.

[CR10] Cooper LJ, Daly KR, Hallett PD, Koebernick N, George TS, Roose T (2018). The effect of root exudates on rhizosphere water dynamics. Proc R Soc.

[CR11] Gregory PJ, McGowan M, Biscoe PV, Hunter B (1978). Water relations in winter wheat 1. Growth of the root system. J Agric Sci.

[CR12] Gregory PJ, McGowan M, Hunter B (1978). Water relations in winter wheat 2. Soil water relations. J Agric Sci.

[CR13] Helliwell JR, Sturrock CJ, Mairhofer S, Craigon J, Ashton RW, Miller AJ, Whalley WR, Mooney SJ (2017). The emergent rhizosphere: imaging the development of the porous architecture at the root-soil interface. Sci Rep.

[CR14] Helliwell JR, Sturrock CJ, Miller AJ, Whalley WR, Mooney SJ (2019). The role of plant species and soil condition in the structural development of the rhizosphere. Plant Cell Environ.

[CR15] Hodgkinson L, Dodd IC, Binley A, Ashton RW, White RP, Watts CW, Whalley WR (2017). Root growth in field-grown winter wheat, some effects of soil conditions, season and genotype. Eur J Agron.

[CR16] Kroener E, Zarebanadkouki M, Bittelli M, Carminati A (2016). Simulation of root water uptake under consideration of nonequilibrium dynamics in the rhizosphere. Water Resour Res.

[CR17] Lilley JM, Kirkegaard JA (2011). Benefits of increased soil exploration by wheat roots. Field Crop Res.

[CR18] Lupton FGH, Oliver RH, Ellis FB, Barnes BT, Howse KR, Welbank PJ, Taylor PJ (1974). Root and shoot growth of semi-dwarf and taller wheats. Ann Appl Biol.

[CR19] Moroke TS, Schwartz RC, Brown KW, Juo ASR (2005). Soil water depletion and root distribution of three dryland crops. Soil Sci Soc Am J.

[CR20] Nelson PN, Banaba M, Scotter DR, Webb MJ (2006). Using soil water depletion to measure spatial distribution of root activity in oil palm (Elaeis guineensis Jacq.) plantations. Plant Soil.

[CR21] Ober ES, Werner P, Flatman E, Angus WJ, Jack P, Smith-Reeve L, Tapsell C (2014). Genotypic differences in deep water extraction associated with drought tolerance. Funct Plant Biol.

[CR22] Passioura JB (1983). Roots and drought resistance. Agric Water Manag.

[CR23] Rabbi SMF, Tighe MK, Flavel RJ, Kaiser BN, Guppy CN, Zhang XX, Young IM (2018). Plant roots redesign the rhizosphere to alter the three-dimensional physical architecture and water dynamics. New Phytol.

[CR24] Read DB, Bengough AG, Gregory PJ, Crawford JW, Robinson D, Scrimgeour CM, Young IM, Zhang K, Zhang X (2003). Plant roots release phospholipid surfactants that modify the physical and chemical properties of soil. New Phytol.

[CR25] Van der Vorst HA (1992). BI-CGSTAB - A fast and smoothly converging variant of bi-cg for the solution of nonsymmetric linear-systems. Siam J Sci Stat Comp.

[CR26] Wasson AP, Richards RA, Chatrath R, Misra SC, Sai Prasad SV, Rebetzke GJ, Kirkegaard JA, Christopher J, Watt M (2012). Traits and selection strategies to improve root systems and water uptake in water-limited wheat crops. J Exp Bot.

[CR27] Wasson AP, Rebetzke GJ, Kirkegaard JA, Christopher J, Richards RA, Watt M (2014). Soil coring at multiple field environments can directly quantify variation in deep root traits to select wheat genotypes for breeding. J Exp Bot.

[CR28] Weir AH, Rayner JH, Catt JA, Shipley DG, Hollies JD (1984). Soil factors affecting the yield of winter wheat, analysis of results from I.C.I. surveys 1979-80. J Agric Sci Camb.

[CR29] Whalley WR, Leeds-Harrison PB, Leech PK, Riseley BA, Bird NRA (2004). The hydraulic properties of the soil at root-soil interface. Soil Sci.

[CR30] Whalley WR, Riseley B, Leeds-Harrison PB, Bird NRA, Leech PK, Adderley WP (2005). Structural differences between bulk and rhizosphere soil. Eur J Soil Sci.

[CR31] Whalley WR, Binley A, Watts CW, Shanahan P, Dodd IC, Ober ES, Ashton RW, Webster CP, White RP, Hawkesford MJ (2017). Methods to estimate changes in soil water for phenotyping root activity in the field. Plant Soil.

[CR32] White R, Kirkegaard JA (2010). The distribution and abundance of wheat roots in a dense, structured subsoil – implications for water uptake. Plant Cell Environ.

[CR33] White CA, Sylvester-Bradley R, Berry PM (2015) Root length densities of UK wheat and oilseed rape crops with implication for water capture and yield. J Exp Bot. 10.1093/jxb/erv077 10.1093/jxb/erv077 PMC4986724 25750427

[CR34] Zarebanadkouki M, Ahmed M, Hedwig C, Benard P, Kostka SJ, Kastner A, Carminati A (2018). Rhizosphere hydrophobicity limits root water uptake after drying and subsequent rewetting. Plant Soil.

[CR35] Zhang XX, Bengough AG, Crawford JW, Young IM (2002). Efficient methods for solving water flow in variably saturated soils under prescribed flux infiltration. J Hydrol.

[CR36] Zhang XY, Pei D, Chen SY (2004). Root growth and soil water utilization of winter wheat in the North China Plain. Hydrol Process.

