# Supplementary material for: A comparison between water uptake and root length density in winter wheat: effects of root density and rhizosphere properties
Source: Plant Soil. 2020 May 1;451(1):345–56. doi: 10.1007/s11104-020-04530-3 (PMC7437669; doi:10.1007/s11104-020-04530-3)
Supplement: Supplementary file 1 — (DOCX 961 kb) [file 11104_2020_4530_MOESM1_ESM.docx]

**A comparison between water uptake and root length density in winter wheat: effects of root density and rhizosphere properties**

**Supplemental data**

XX Zhang^1^, PA Whalley^2^, RW Ashton^1^, J Evans^1^, MJ Hawkesford^1^, S. Griffiths^3^, ZD Huang^4^ H Zhou^5,6^, SJ Mooney^5^ and WR Whalley^1^

^1^Rothamsted Research, Harpenden, Hertfordshire, AL5 2JQ.

^2^ University of Oxford, Radcliffe Observatory, Andrew Wiles Building, Woodstock Rd, Oxford OX2 6GG

^3^John Innes Centre, Norwich Research Park, Norwich, NR4 7UH, UK

^4^ Farmland Irrigation Research Institute, Chinese Academy of Aricultural Sciences, Xinxiang 453002, Henan, China.

^5^ School of Biosciences, University of Nottingham, Sutton Bonington Campus, Loughborough, Leicestershire LE12 5RD, United Kingdom

^6^ State Key Laboratory of Soil and Sustainable Agriculture, Institute of Soil Sciences, Chinese Academy of Sciences. 71 East Beijing Road, Nanjing 210008, P.R. China

Lines used in this study

|  | Name | Plot number | | | BTK Code | Genotype | Allele |
| --- | --- | --- | --- | --- | --- | --- | --- |
| 1 | nil1-1 | 65 | 188 | 461 | 1 | 141-16-10P-2DEM(4-2) | p |
| 2 | nil1-10 | 91 | 127 | 378 | 10 | 141-7-11W-2DYLD(6-13) | w |
| 3 | nil1-100 | 183 | 201 | 380 | 100 | 729-77-2W-5AEM(6-24) | w |
| 4 | nil1-101 | 25 | 43 | 96 | 101 | 729-77-4P-5AEM(6-30) | p |
| 5 | nil1-102 | 171 | 281 | 463 | 102 | 729-77-6W-5AEM(6-25) | w |
| 6 | nil1-12 | 230 | 262 | 332 | 12 | 141-7-13P-7BHT(5-12) | p |
| 7 | nil1-14 | 84 | 243 | 272 | 14 | 141-7-16P-2DYLD(6-18) | p |
| 8 | nil1-17 | 191 | 336 | 480 | 17 | 141-7-17P-7BHT(5-13) | p |
| 9 | nil1-19 | 319 | 455 | 501 | 19 | 141-7-18W-7BHT(5-7) | w |
| 10 | nil1-2 | 17 | 202 | 269 | 2 | 141-16-12W-2DEM(5-16) | w |
| 11 | nil1-20 | 23 | 106 | 225 | 20 | 141-7-19P-2DYLD(6-20) | p |
| 12 | nil1-22 | 126 | 355 | 385 | 22 | 141-7-2W-2DYLD(6-8) | w |
| 13 | nil1-25 | 135 | 194 | 333 | 25 | 141-7-5P-7BHT(5-8) | p |
| 14 | nil1-28 | 123 | 282 | 354 | 27 | 141-7-6P-7BHT(5-9) | p |
| 15 | nil1-29 | 38 | 182 | 261 | 29 | 141-7-7P-7BHT(5-10) | p |
| 16 | nil1-33 | 160 | 239 | 409 | 33 | 209-38-15P-6AHT(4-16) | p |
| 17 | nil1-35 | 99 | 122 | 134 | 35 | 209-38-1W-6AHT(4-6) | w |
| 18 | nil1-43 | 8 | 72 | 404 | 43 | 209-57-17W-3AEM(3-7) | w |
| 19 | nil1-44 | 76 | 138 | 151 | 44 | 209-57-18P-3AEM(3-14) | p |
| 20 | nil1-45 | 20 | 348 | 371 | 45 | 209-57-19W-3AEM(3-11) | w |
| 21 | nil1-48 | 41 | 343 | 365 | 46 | 209-57-1P-3AEM(3-12) | p |
| 22 | nil1-53 | 278 | 345 | 351 | 53 | 292-69-10P-6AHT(2-3) | p |
| 23 | nil1-55 | 79 | 364 | 437 | 54 | 292-69-13W-6AHT(3-17) | w |
| 24 | nil1-64 | 176 | 198 | 213 | 64 | 34-19-11P-2BEM(6-7) | p |
| 25 | nil1-65 | 234 | 296 | 423 | 65 | 34-19-12W-1BEM(6-6) | w |
| 26 | nil1-66 | 114 | 337 | 459 | 66 | 352-54-11W-5AHT(2-18) | w |
| 27 | nil1-69 | 129 | 155 | 167 | 69 | 352-54-18W-5AHT(2-17) | w |
| 28 | nil1-70 | 190 | 223 | 289 | 70 | 352-54-1W-5AHT(2-16) | w |
| 29 | nil1-71 | 169 | 235 | 473 | 71 | 352-54-20P-5AHT(1-3) | p |
| 30 | nil1-72 | 70 | 347 | 353 | 72 | 352-54-22W-3AEM(2-11) | w |
| 31 | nil1-73 | 34 | 287 | 472 | 73 | 352-54-3P-3AEM(2-12) | p |
| 32 | nil1-79 | 279 | 315 | 499 | 79 | 468-69-15W-6AHT(1-11) | w |
| 33 | nil1-80 | 74 | 137 | 406 | 80 | 468-69-17P-6AHT(6-21) | p |
| 34 | nil1-85 | 21 | 471 | 490 | 85 | 468-69-22P-6AHT(6-23) | p |
| 35 | nil1-86 | 346 | 470 | 489 | 86 | 468-69-2P-6AHT(1-16) | p |


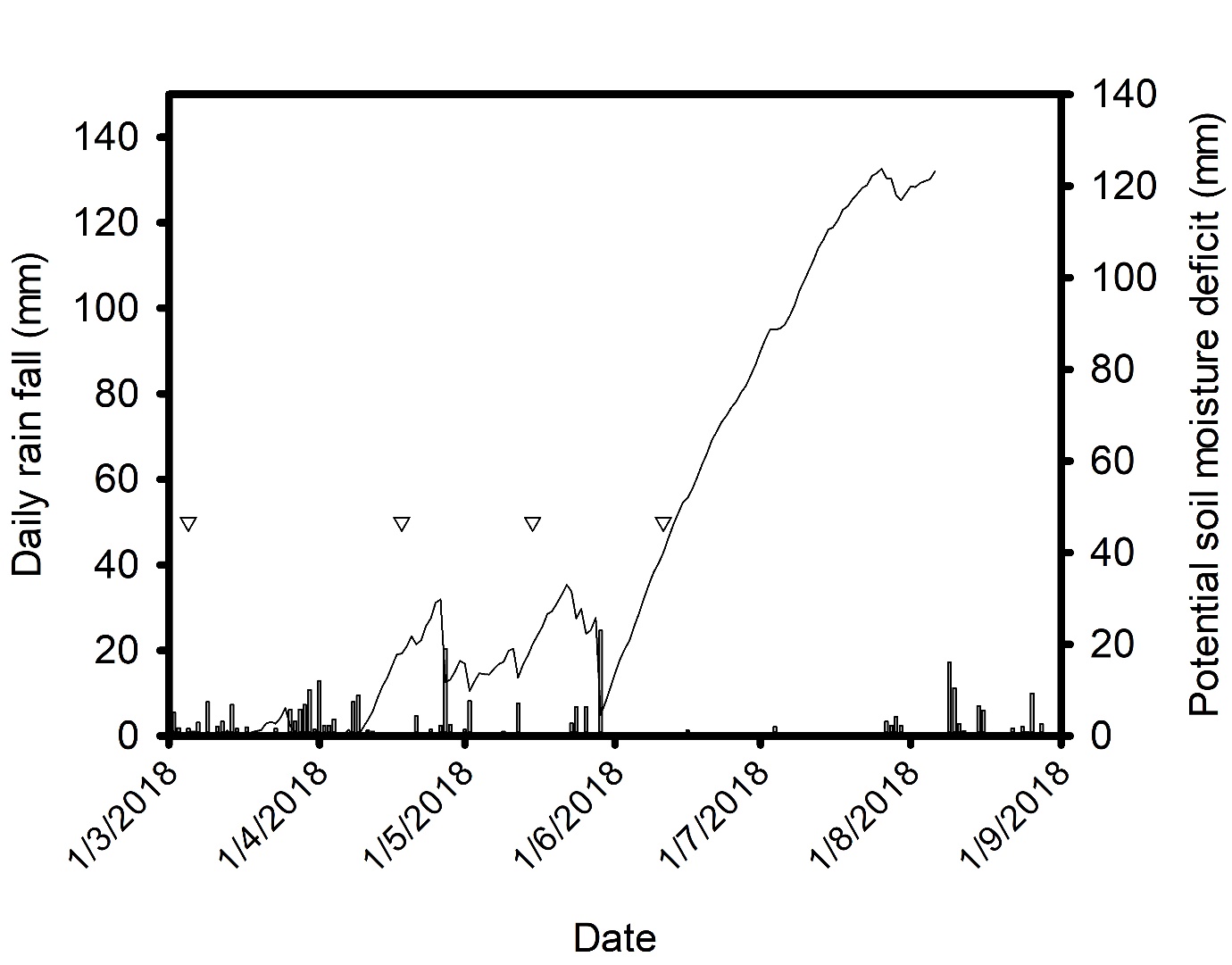


FIGURE S1. Patterns of rainfall and soil moisture deficit in the 2017/2018 winter wheat season. The measurement points of soil moisture monitoring are shown by the open triangles.


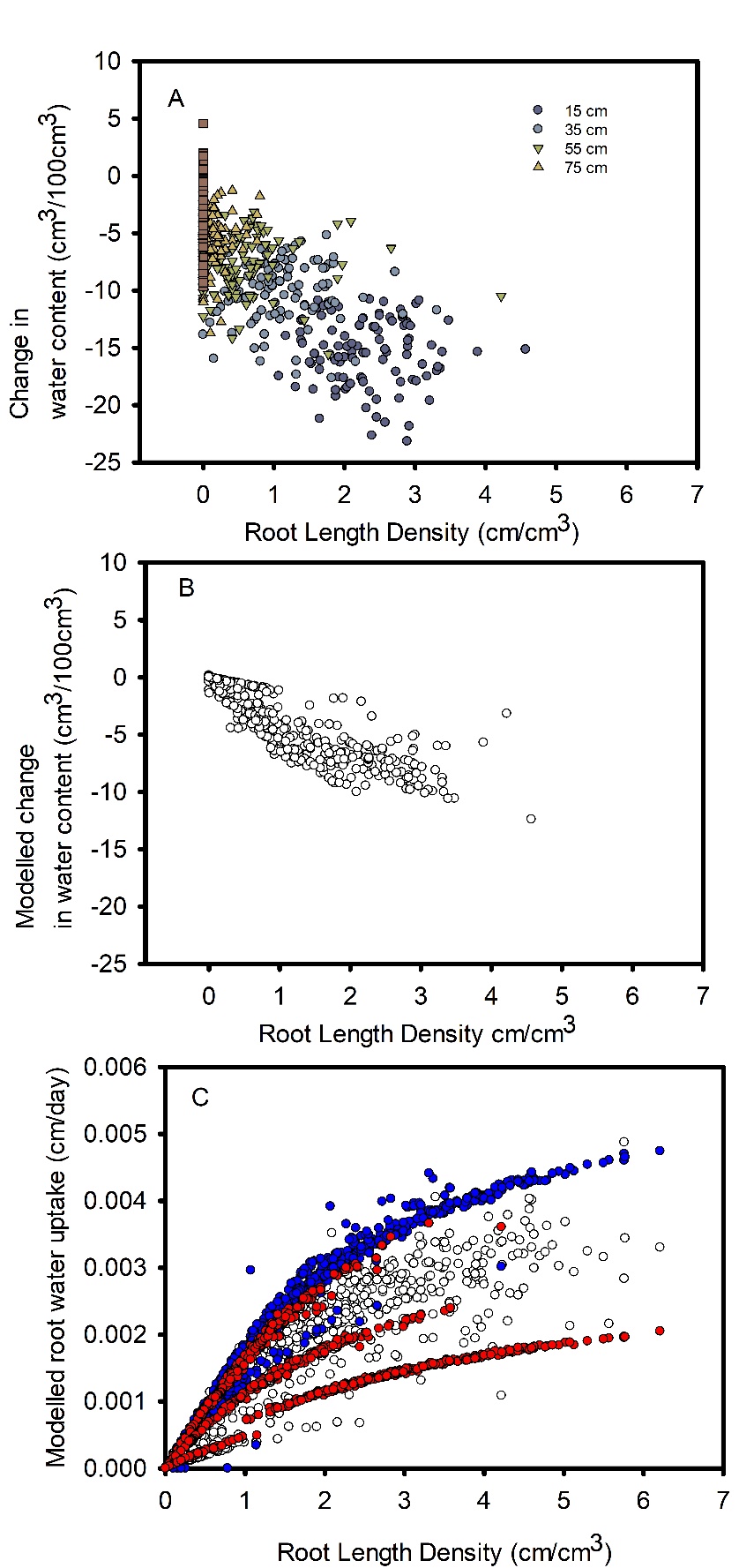


FIGURE S2. In panel A the measured drying of soil is plotted against root length density. In panel B an adjust water content that takes account of vertical flow is plotted against root length density. Comparisons of panels A and B shows that the change in water content at very low root length densities is related to either drainage in the deeper layers or evaporation in the shallow layers. Panel C shows the modelled root water uptake plotted against root length density for the three simulations: blue symbols for the initially wet profile, open symbols for the actual water profile and red symbols for the initially dry profile.


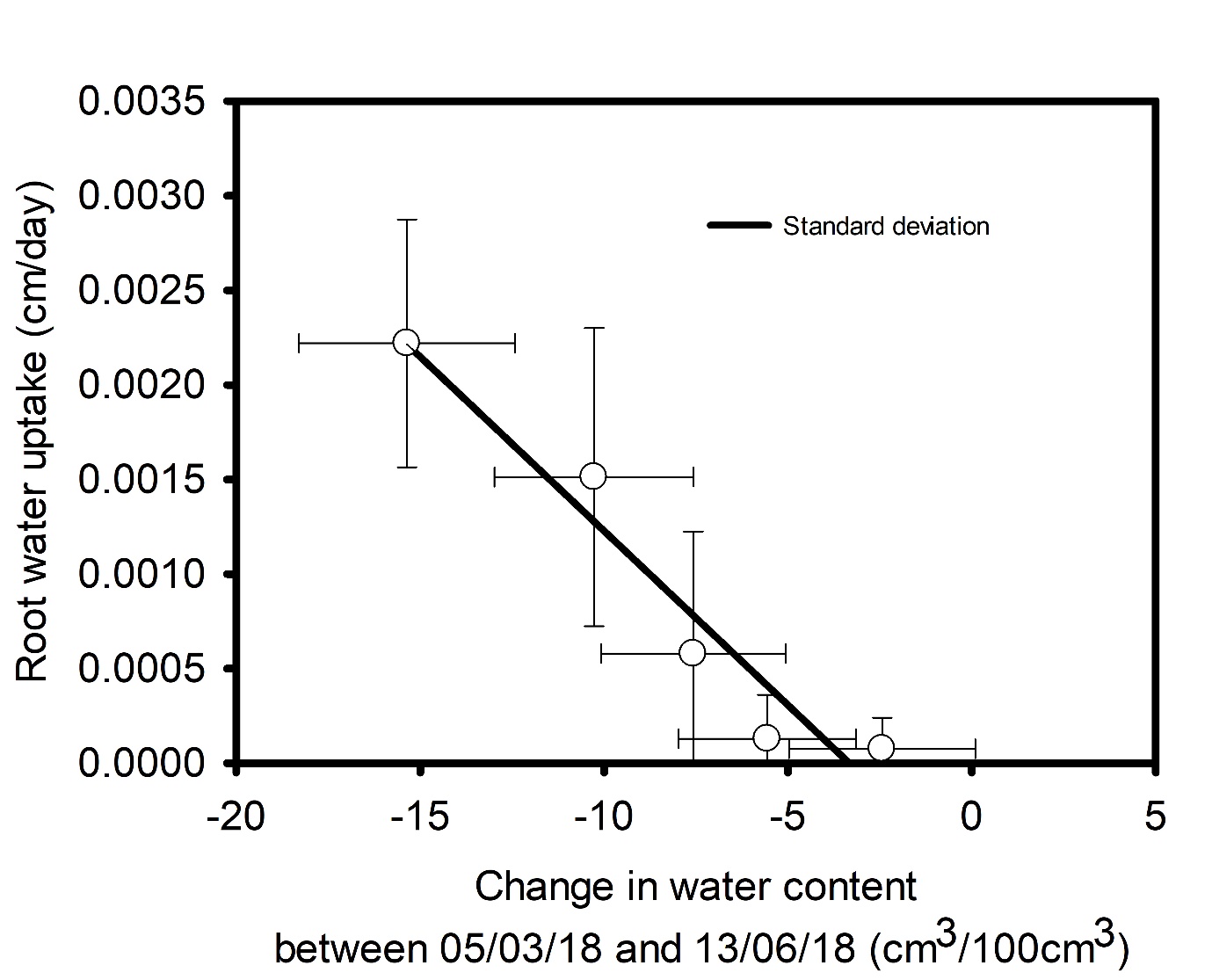


FIGURE S3. The respective predictions of root water uptake from the model plotted against the mean change in water content at depths of 15, 35, 55, 75, and 95 cm. The standard deviation of the mean is indicated. The regression explains 38 percent of the variance in the data (P<0.001).


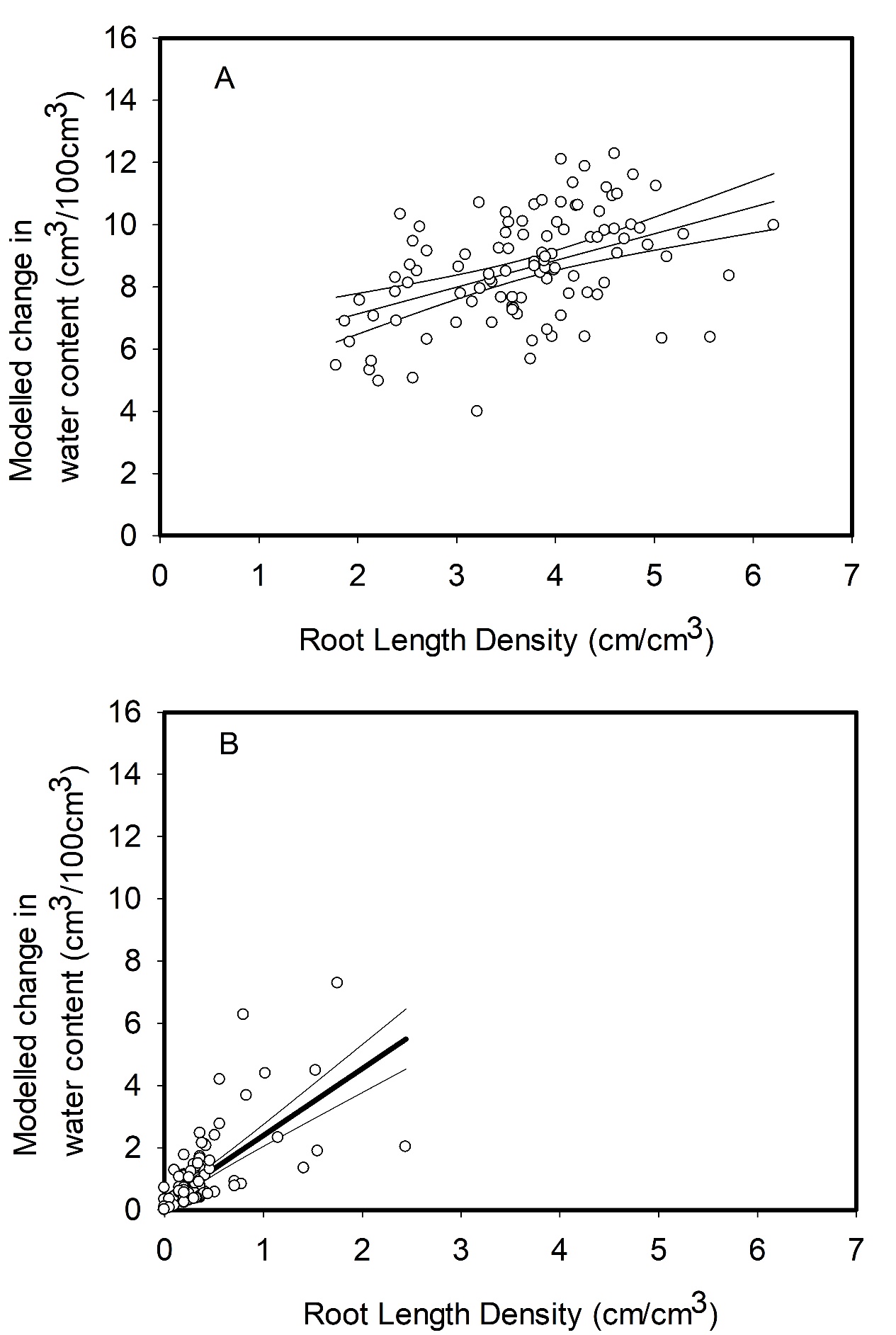


FIGURE S4. Modelled change in water content plotted against root length density at depths of 5 cm (A) and 65 cm (B). In the shallow, 5 cm, layer only 21.3% of soil drying is explained by root length density whereas at 65 cm, 46.4% of the change in water content is explained by root length density.
